# Supplementary material for: A Synthetic Platform for Antibody Junctional Diversification Beyond Natural Constraints
Source: Adv Sci (Weinh). 2026 Aug 21;13(63):e77339. doi: 10.1002/advs.77339 (PMC13496277; doi:10.1002/advs.77339)
Supplement: Supplementary file 1 [file ADVS-13-e77339-s001.docx]

Supporting Information

A Synthetic Platform for Antibody Junctional Diversification Beyond Natural Constraints

Wang Liu, Jun Yang, Yuhang Zhang*, Xiaoli Xue *

W. Liu, J. Yang, X. Xue

State Key Laboratory of Microbial Metabolism, and School of Life Sciences & Biotechnology

Shanghai Jiao Tong University

Shanghai, 200240, P. R. China
E-mail: xlxue@sjtu.edu.cn

Y. Zhang
Sheng Yushou Center of Cell Biology and Immunology, School of Life Sciences and Biotechnology

Shanghai Jiao Tong University

Shanghai, 200240, P. R. China

E-mail: yuhang.zhang@sjtu.edu.cn

**Figure S1. Defining the essential requirements for V-J recombination in HEK293T cells, related to Figure 2.**

(**A**) Schematic of mutant constructs assessing essential factors for V-J rearrangement in HEK293T cells. The Wild-Type (WT) group contains functional 12/23 RSS (heptamer in blue) and RAG1. The 12/23 RSS^mut^ group carries a heptamer mutated to AAAAAAA (red), disrupting RAG recognition. The RAG^mut^ group expresses a catalytically inactive RAG1 (D708A) to abrogate recombination activity.

(**B**) Flow cytometric analysis of recombination efficiency. HEK293T cells were transfected with the EGFP reporter plasmid along with different RAG combinations: (i) wild-type RAG1/RAG2(WT), (ii) RSS heptamer mutant (12/23RSS^mut^), and (iii) RAG1 catalytic dead mutant (RAG^mut^).

**Figure S2. Assessment of HMGB1 co-expression on RAG-mediated recombination efficiency, related to Figure 2.**

(**A**) Atomic model of the RAG-RSS-HMGB1 complex (based on PDB:6CG0).

(**B**) Western blot analysis of light-chain. HEK293T cells were co-transfected with RAG1/2 expression constructs alongside either an empty vector control or an HMGB1 expression plasmid. Recombination efficiency was evaluated by Western blot detecting FLAG. β-tubulin served as a control.

**Figure S3. Construction and recombination of stable-integration HEK293T carrying antibody gene cassettes, related to Figure 2.**

(**A**) Schematic of the workflow for generating stable cell lines and subsequent antibody gene reprogramming. The diagram illustrates the construction and selection of stable cell lines using the piggyBac transposon system, followed by antibody gene reprogramming via co-transfection of a RAG1 expression plasmid and a truncated RAG2Δ mRNA. The final step involves enrichment of antibody-expressing cell populations by blasticidin S selection.

(**B**) Analysis of antibody gene integration efficiency mediated by the piggyBac transposon system. Flow cytometry analysis showing the percentage of mCherry-positive HEK293T cells after co-transfection with a piggyBac-mCherry reporter plasmid and a transposase expression plasmid, followed by puromycin selection for varying durations.

(**C**) qPCR analysis of antibody gene copy number in the HBSAb light chain-stable integrating HEK293T.Primers were designed specificly binding to constant region and RSS insertion of light chain coding gene. The absolute copy number per cell was calculated using a standard curve, with the ACTB gene serving as the internal reference control.

(**D**) Western blot analysis of antibody gene rearrangement products in HEK293T cells. Target antibody genes were delivered through either transient plasmid transfection (lane 1) or stable cell line generation (lane 2), followed by co-expression of RAG1 and RAG2.

(**E**) PCR-based assessment of RAG1/2-mediated antibody gene rearrangement under optimized conditions. The purple arrows indicate the locations of the primers. Lane M: DNA ladder; Lane 1: Pre-optimization control; Lane 2: Stage 1 optimization (RAG2 truncation mutant + mRNA delivery); Lane 3: Stage 2 optimization (stable transfection + antibiotic selection); Lane 4: Negative control (no RAG1/2).

(**F**) Bar graph summarizing the optimized efficiency of RAG1/2-mediated antibody gene rearrangement, as quantified from the data shown in panel (**E**).

**Figure S4. Mammalian cell surface display and flow cytometric analysis of Fab and IgG antibodies**

(**A**)Immuno-fluorescence analysis of antibody surface display and antigen binding. Immunofluorescence staining of cells demonstrating the surface display and functionality of engineered antibodies. Panels show: a negative control transfected with empty pDisplay plasmid (top), successful display and HBSAg-binding of Fab antibodies (middle) and full-length IgG antibodies (bottom). Red fluorescence indicates the myc tag labeled with an AF647-conjugated antibody, green fluorescence shows FITC-conjugated HBSAg antigen, and blue represents DAPI-stained nuclei. The co-localization of red and green signals on the cell membrane confirms successful antibody display and specific antigen-binding capability. Scale bar:10μm.

(**B**) Flow cytometric analysis of surface display efficiency. Pseudocolor dot plots depict the surface expression of the myc-tagged antibodies in cells transfected with empty pDisplay plasmid (left), Fab‑displaying construct (middle), or IgG‑displaying construct (right).

(**C**) ARESEC-mediated reprogramming yields functional light chains that successfully assemble into antigen-binding Fab (top) and IgG (bottom) antibody formats, both of which demonstrate specific recognition of HBSAg.

**Figure S5. Nucleotide Diversification Profile of CDRL 1/2/3 by High-Throughput Sequencing**

High-throughput sequencing analysis of nucleotide-level diversification across CDRL1, CDRL2, and CDRL3 of HBSAg antibody. The heatmap shows log₁₀-transformed values of mutation frequency. Scissors and dashed lines indicate RAG1/2 recognition and cleavage sites.

**Figure S6. Reprogramming of Nivolumab by the ARESEC Platform, related to Figure 5.**
(**A**) PCR analysis of the rearrangement efficiency in CDRL1, CDRL2, and CDRL3 regions.

(**B**) Western blot analysis confirming light chain protein expression after reprogramming.

(**C**) Top 12 most abundant amino acid sequences and their mutation types in Nivolumab following rearrangement by ARESEC. The percentage to the left of each sequence indicates the proportion of reads in the DNA next-generation sequencing data. The sequences are color-coded: red boxes for antibody light chain–antigen interaction sites. gray circles for the reference sequence, yellow circles for deletions, blue circles for insertions, and purple circles for amino acid point mutations.

(**D**) Flow cytometric sorting of double-positive cell populations for antibody display and antigen binding following ARESEC-mediated reprogramming of Nivolumab.

**Figure S7. Analysis of integrity, purity, and aggregation state of Nivolumab and its reprogrammed variants after purification, related to Figure 5.**

(**A**) SDS-PAGE analysis under reducing conditions. Lane M: Protein molecular weight marker; Lanes N0-N3: Purified antibody samples, showing bands at approximately 50 kDa (Heavy Chain) and 25 kDa (Light Chain).

(**B**) SDS-PAGE analysis under non-reducing conditions. Lane M: Protein molecular weight marker; Lanes N0-N3: Purified antibody samples, showing a single major band at approximately 150 kDa, corresponding to the intact IgG molecule.

(**C**) Purity of IgG, analyzed by Size-exclusion chromatography (SEC-HPLC).

**Figure S8. Reprogramming of Durvalumab by the ARESEC Platform, related to Figure 6.**

(**A**) PCR analysis of the rearrangement efficiency in CDRL1, CDRL2, and CDRL3 regions.

(**B**) Western blot analysis confirming light chain protein expression after reprogramming.

(**C**) Sequencing analysis of the distribution and frequency of nucleotide mutation types at each position in CDRL1, CDRL2, and CDRL3 following Durvalumab rearrangement by ARESEC. The heatmap shows log₁₀-transformed values of mutation frequency. Scissors and dashed lines indicate RAG1/2 recognition and cleavage sites.
